# Optimization of automotive HVAC performance and passenger comfort through intelligent algorithms

**DOI:** 10.1007/s10973-026-15752-5

**Published:** 2026-06-17

**Authors:** Ali Hussein Abdulkarim, Andaç Batur Çolak, Ali Ates, Mohammed Alharbi, Yakup Karakoyun, Ahmet Selim Dalkilic

**Affiliations:** 1 Department of Mechanical Engineering, College of Engineering, University of Kirkuk, Kirkuk, 36001 Iraq; 2 Department of Information Systems and Technologies, Faculty of Computer and Information Sciences, Niğde Ömer Halisdemir University, 51240 Niğde, Türkiye; 3 Department of Mechanical Engineering, Faculty of Engineering and Architecture, Sinop University, 57000 Sinop, Türkiye; 4 Mechanical Engineering Department, College of Engineering and Architecture, Umm Al-Qura University, Makkah, Saudi Arabia; 5 Department of Mechanical Engineering, Van Yuzuncu Yil University (YYU), Engineering Faculty, 65080 Van, Türkiye; 6 Department of Mechanical Engineering, Mechanical Engineering Faculty, Yildiz Technical University (YTU), 34349 Istanbul, Türkiye; 7 Scientific Research Department, Azerbaijan University of Architecture and Construction (AzUAC), AZ1073 Baku, Azerbaijan

**Keywords:** Automotive, HVAC, Artificial neural networks, Thermal comfort, Predictive modeling

## Abstract

Optimizing passenger thermal comfort while maintaining energy efficiency remains a critical challenge in automotive climate control. This study investigates the complex thermal interaction between a passenger and a car seat, introducing a novel technique for direct thermal management through the vehicle’s heating, ventilation, and air-conditioning system. An experimental setup was developed using evaporator coils integrated beneath seat surfaces, addressing a significant gap in the literature regarding two-way (heating and cooling) seat thermal management. Artificial Neural Networks were employed to model the ambiguous system parameters and to predict thermal performance. The results show an excellent predictive accuracy with Mean Squared Error of 1.29E-02 and correlation coefficient of 0.99659. The average deviations were under −0.21% for cooling capacity, −0.02% for heating capacity and 0.38% for coefficient of performance. These results show that the integration of intelligent algorithms into modified heating, ventilation, and air conditioning architectures significantly improves occupant comfort and system efficiency, thereby providing a powerful data-driven framework for next-generation automotive climate control solutions.

## Introduction

By eliminating the need for physical modeling and improving the methodology of experiments data-driven approaches prove to be a more effective solution [1]. Statistical correlations between temperature and pressure readings have long been used by automotive climate control systems to evaluate system performance [2]. Artificial Intelligence (AI), especially Machine Learning (ML), provides a new way to understand the dynamical behavior of these systems and produce accurate predictions [3]. Calibration of human comfort thermal systems can be difficult and time consuming especially for complicated systems like automotive HVAC [4]. Startups encounter many issues such as the absence of large-labeled datasets and issues with model transferability that hinder the translation of AI development to low-cost solutions [5]. For forecasting system behavior mathematical representations such as inverse models and forward simulation are crucial [6]. These models fall into two categories: mechanistic and statistical [7].

To guarantee passenger comfort automotive climate control systems have historically depended on statistical relationships between temperature and system performance. AI, more especially ML, has become a potent instrument for comprehending the intricate dynamics of these systems and producing accurate predictions [8, 9]. The ability of MLs to find patterns from data and develop predictive models increases the possibility of controlling thermal comfort in cars. The integration of state-of-the-art technologies such as ML and AI has revolutionized many industries in recent years leading to innovation and optimization across diverse domains. The automotive and energy sectors are among the most impacted, as they have experienced significant advances in areas such as energy management and thermal comfort systems. AI and ML have completely revolutionized the prediction and control of thermal comfort in automotive applications, and now offer individualized climate control settings that, simultaneously, improve passenger comfort and energy efficiency. Today, AI-enabled systems can dynamically forecast and adjust cabin temperatures based on real-time information including passenger preferences, vehicle speed and outside weather conditions. Similarly, the energy industry has adopted AI and ML methods to improve energy management systems and raise the effectiveness of energy distribution and consumption.

In modeling the thermal comfort of car seats and cabins researchers have made great progress [10, 11]. Sensitive parts of the body, including the shoulders, back arms and buttocks have been found to be extremely sensitive to temperature changes by examining the effects of seat cooling systems on passenger comfort [4]. Successful applications of ML and statistics have been made in electric vehicles and other domains to optimize energy consumption, improve passenger comfort and maintain optimal battery temperatures [12]. Data on driving behavior and the particulars of heat pump systems are considered in these models. Using Computational Fluid Dynamics (CFD) the authors also helped develop the compartment simulation [13, 14]. Ju & Co. [3] created a machine-learning-based method for forecasting individual thermal comfort in automobile cabins which can differ greatly among people because of various physiological and psychological aspects. The study concentrated on using AI to examine ambient conditions and seat-heating patterns in authentic winter settings. Based on sensor data from cars, the authors tested several ML algorithms including support vector machine and kNN to forecast individual comfort. According to the findings ML could predict passenger heating preferences up to 96% of the time. This suggests that automated HVAC system adjustments could improve passenger comfort without requiring human input. Another study by Basciotti et al. [4] aimed to reduce energy consumption by optimizing the thermal management systems in electric vehicles. For batteries and other car parts the authors proposed a novel method that uses refrigerant-based heating and cooling systems. The study demonstrated that the deployment of these systems could greatly increase vehicle efficiency and range particularly in instances of extreme temperature. The dual benefits of thermal management in electric vehicles were demonstrated by the integration of energy-efficient HVAC systems which reduced the energy drawn from the vehicles’ main power source while simultaneously improving passenger comfort. Likewise, the authors in [11] investigated the use of AI to control the heat loads in cars emphasizing ML methods to forecast passenger’s thermal preferences. The study trained algorithms that could predict the comfort levels of individual passengers by combining physiological measurements with environmental data such as the outside temperature and humidity. The findings suggested that by instantly adjusting to user preferences customized climate control systems could greatly increase passenger satisfaction and energy efficiency. König et al. [15] also investigated how AI and sophisticated sensor technologies might be used in smart buildings for energy management. It concentrated on creating smart HVAC systems that adjust to internal and external climate conditions to maximize energy efficiency. The study demonstrated how well ML algorithms predict energy needs and modify HVAC operations in real-time saving a significant amount of energy without sacrificing occupant comfort. Although data-driven models offer insightful information it can be difficult to choose the best one. Comparing the performance of various models is frequently done through statistical and ML analyses [16, 17]. There is no surefire method that consistently performs better than others even though some models show higher reliability [18, 19]. It can be advantageous to combine statistical and ML models, but it is essential to test different models on datasets and make sure they work consistently with data that haven’t been seen yet. Recent research has investigated model stability and used cutting-edge strategies like synthetic data generation. Additionally, the versatility and complexity of ML models offer encouraging opportunities to enhance the regulation of seat and cabin thermal comfort. Through the integration of statistical techniques, mathematical equations and experimental research, we can examine and comprehend the intricate relationships between equipment configurations and operational influences. Except for the authors’ earlier publication [20] which examines heating and cooling a vehicle seat using such coils the literature survey found no experimental studies on the two-way (heating and cooling) use of vehicle air conditioner heat exchanger coils embedded in car seats. The primary objective of this research is to experimentally evaluate and numerically model a novel two-way automotive seat thermal management system integrated directly into a modified vehicle HVAC architecture. Specifically, this study aims to analyze the thermal interaction between a seated occupant and the seat surface while developing a robust artificial neural network (ANN) framework to accurately predict key performance parameters, namely cooling capacity (*Q̇*_H_​), heating capacity (*Q̇*_H_​), and the coefficient of performance (COP) across a wide range of operational conditions. To predict important parameters like *Q̇*_L_ *Q̇*_H_, and the COP an ANN, a model was created using the data from the study. The data collected were used to train the ANN model. These data included temperature fluctuations, the system’s reaction to various conditions, and the distribution of heat throughout the vehicle seat. The model was developed to learn intricate patterns and relationships between variables using these data which enables it to predict heat transfer rates during both heating and cooling modes with accuracy. A more accurate assessment of the system’s energy efficiency was also made possible by the COP prediction which aided in striking a balance between energy consumption and thermal comfort. The development of cutting-edge energy-efficient automotive heating and cooling systems can be greatly aided by the developed ANN model in the current investigation.

## Methodology

**Fig. 1:**
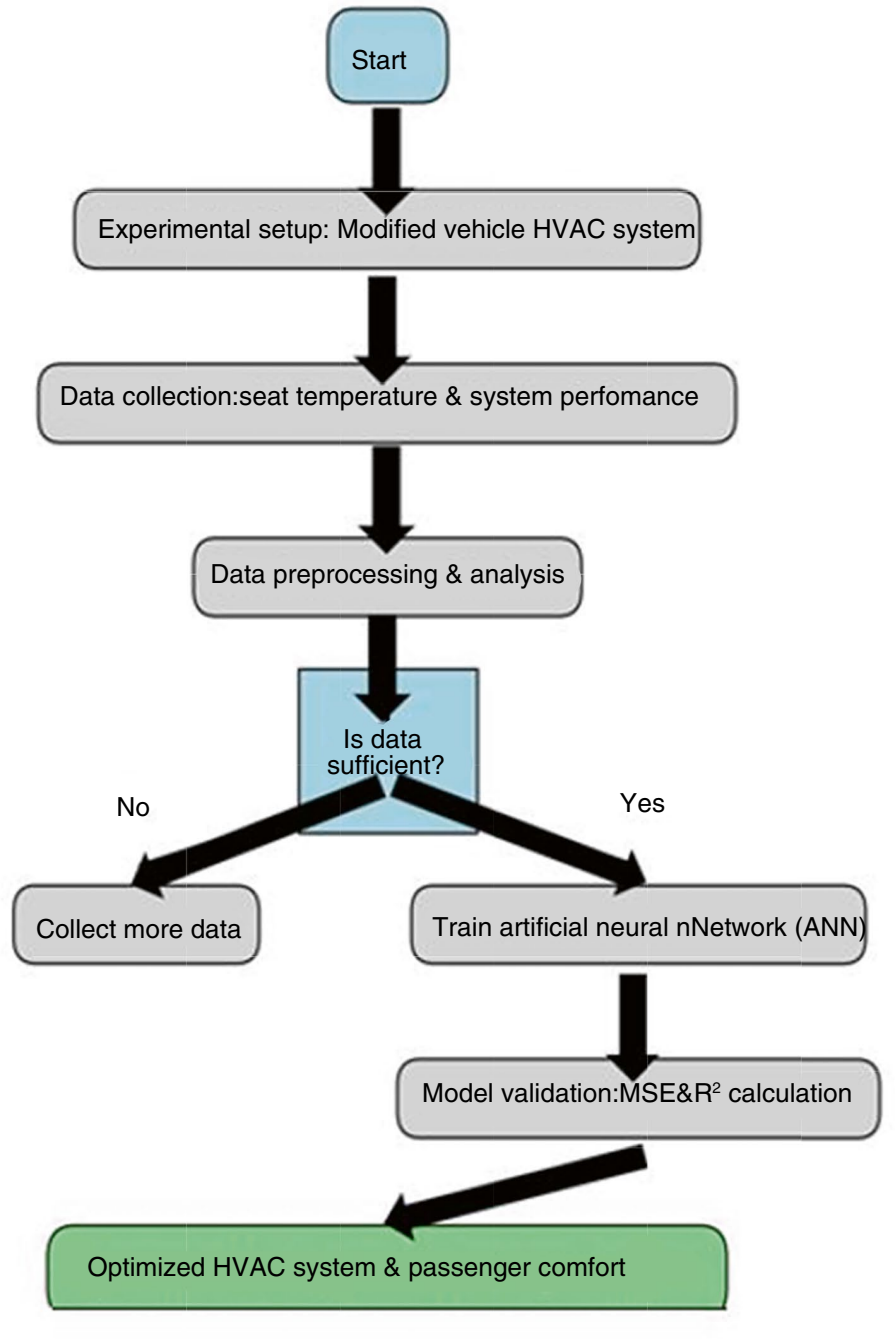
Flowchart of methodology for optimizing automotive HVAC system and passenger comfort using ANNs

To facilitate data collection, a modified automotive HVAC system is meticulously set up as an experimental environment. An array of sensors is positioned strategically throughout this system to monitor several parameters such as airflow seat surface temperatures and the HVAC systems overall performance. Throughout the experiment, these sensors continuously collect data documenting the thermal dynamics of the vehicle cabin as it reacts to various operating circumstances. To ascertain whether the data are sufficient for training the ANN model, its sufficiency is assessed after it has been collected. To make sure the dataset can appropriately depict the intricate relationships within the system, this decision-making process examines data coverage variability and completeness. Additional data are gathered to fill in any gaps in the dataset if it is discovered to be insufficient in volume or diversity. This ensures that the model has enough data to work with. The robustness of the ANN models training and eventual predictive capabilities are ensured by this iterative process of data collection and evaluation which continues until the dataset is judged comprehensive and reliable. The next step is to train an ANN model after enough data have been collected as indicated by the right branch in Fig. 1. The purpose of this model is to forecast and optimize important HVAC system parameters such as energy consumption air flow control and temperature regulation. By using the gathered data to find patterns and connections within the system, the ANN model can simulate and predict the HVAC system’s performance under different circumstances. In an iterative training process, the model continuously modifies its internal masses to reduce prediction errors and boost accuracy. The model’s efficacy is assessed using several performance metrics. The mean squared error (MSE) which calculates the average squared difference between expected and actual values is one of the important metrics. The more accurate the model’s predictions are the lower the MSE. The reliability of the model is also clearly indicated by the correlation coefficient (*R*) which is used to evaluate how well the predictions of the model match the actual results. The precision of the model is further validated by a higher *R* value which indicates that it accounts for a sizable amount of data variability. An important development in the architecture and functioning of automotive HVAC systems is represented by this methodical approach that combines data-driven methodologies with ML techniques. The system is better able to manage intricate thermal dynamics, maximize resource utilization and enhance user comfort by utilizing AI-based models which will ultimately result in more intelligent and sustainable climate control solutions for contemporary automobiles.

### Experimental investigation

**Fig. 2:**
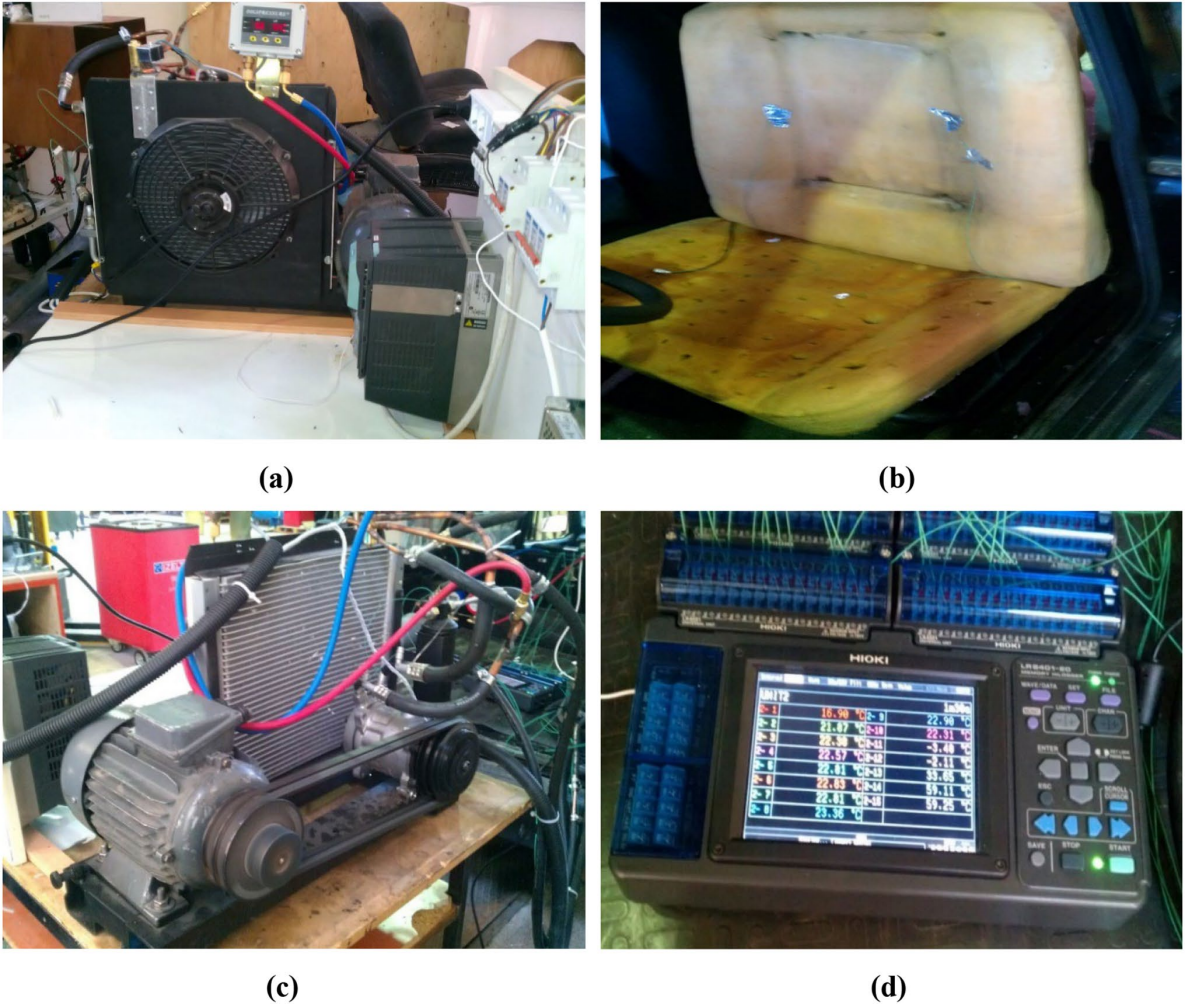
The experimental setup for automotive HVAC optimization: **a** Cooling unit with fan, **b** Car seat with HVAC coils, **c** Compressor unit, **d** Data logger monitoring temperature readings for automotive HVAC system optimization

The testing enclosure was a Renault Toros SW passenger vehicle with an air conditioner that does not use refrigerant. The vehicle’s air conditioning system used an electric motor to power the compressor rather than the vehicle’s engine. However, modifications to both the HVAC system and the seat were required to enable data collection for thermal calculations, as illustrated in Fig. 2.

The stainless steel corrugated (spiral) hoses with the outer diameter of 2.5 mm were designed in a serpentine configuration and implemented in the seat cushion and backrest sections. The mechanical durability of these hoses was better than that of polymer or silicone alternatives and they also had relatively higher thermal conductivity, which is why they were preferred. Copper tubes could have been chosen because of their higher thermal conductivity, but their mechanical durability would be much lower under repeated deformation conditions. The thermal conductivity of the stainless-steel corrugated hoses used in this study is about 22–23 W m^−1^ K^−1^. These hoses were underneath the seat upholstery. To improve heat spreading, a thin layer of aluminum foil was placed underneath the serpentine structure, and the top surface was covered with an approximately 1 mm thick silicone fabric layer. The silicone layer slightly reduces the overall thermal performance but offers a good compromise for passenger comfort.

Calibration of the thermocouples was done prior to every experiment run to enhance accuracy of measurements. To observe the thermal characteristics of the system, 23 thermocouples in various locations were installed. There were five thermocouples on the seat back surface and five on the seat cushion surface to measure the spatial temperature distribution on the seat. The rest of the thermocouples were also installed at the inlet and outlet of the key components of the HVAC such as the compressor, expansion part, and the heat exchanger part, to monitor thermal changes at both refrigerant and system levels. Other measurements made comprised compressor inlet and outlet pressure and compressor rotational speed. This instrumentation design was able to measure the temperature of the seat surface and the running parameters of the HVAC at the same time in every test. A total of five thermocouples were strategically placed on the surfaces of the back seat and cushion, in addition to two more thermocouples on the distributor and collector manifolds. A total of 23 temperature measurements were taken from various locations throughout the system, including the input and output of components such as the compressor, expansion valve, and heat exchanger.

To provide stable and controllable operating conditions, the compressor of the automotive air-conditioning system was driven by an electric motor instead of the vehicle engine. Compressor speed was regulated by a frequency inverter, which allowed the operating conditions to be reproduced independently of engine fluctuations. The seat coil circuit was operated in either cooling or heating mode through a four-way valve, so that the same seat-based thermal system could function as an evaporator or a condenser depending on the selected mode. The pressures at the inlet and the output were measured, as well as the velocity of the compressor. A four-way valve was used to switch the function of the seat coil between the evaporator and condenser modes. Heating and cooling experiments were conducted under cold and hot weather conditions respectively, with no influence of direct sunshine or engine heat. A compressor driven by an electric motor was used to simulate normal engine conditions and was controlled by a frequency inverter. The compressor consumed approximately 10% of the total engine power. Testing was conducted on mannequins. Before each test the system was carefully checked for any possible leaks and the thermocouples were calibrated. Temperature data was collected at a frequency of 0.2 Hz, which corresponds to measurements every 5 s. Initially, the air conditioner was operated at idle for 5 min and then the speed of the compressor was changed, and it was operated for 10 min until it reached stable condition. Then temperature readings started and took five minutes. A manikin was placed in the chair for 5 min and the recording continued for an additional 5 min after it left. Thermal comfort of the manikin was further evaluated. The experiments were carried out at 12 different compressor speeds, ranging from 500 to 1600 rpm with a step of 100 rpm. This experimental setup is based on our previous work on heating and cooling vehicle seats [20].

The experiments were carried out under cooling and heating conditions. Cooling tests were carried out during hot weather, while heating tests were performed during cold weather. To reduce external disturbances, the tests were performed without direct solar radiation and without the thermal influence of engine operation. In this way, the measured thermal response was primarily governed by the HVAC-seat system itself. The compressor was tested at 12 operating speeds ranging from 500 to 1600 rpm with increments of 100 rpm. This range allowed the effect of compressor speed on seat surface temperature and system COP to be systematically evaluated. The detailed instrumentation, controlled boundary conditions, and repeatable test sequence ensured that the generated dataset was suitable for reliable thermodynamic evaluation and data-driven prediction of system performance.

Extensive instrumentation, controlled boundary conditions and repeatable test sequence ensured the generated dataset is suitable for reliable thermodynamic evaluation and data-driven prediction of system performance. The automotive HVAC system is assumed to provide a constant cooling (or heating) capacity within the specified operating conditions. Therefore, potential variations in system performance under high rotational speeds or severe operating conditions have been neglected. COP exhibit diminishing returns at higher rpm despite increased airflow, due to the dominant energy loss mechanisms. COP shows diminishing returns at higher compressor rpm because the gain in heat exchange and airflow distribution is eventually offset by the faster rise in power consumption. At low to moderate rpm, increasing speed improves refrigerant circulation and thermal transport, which can enhance system performance. However, at higher rpm, the compressor requires greater mechanical work, fan-assisted heat transfer no longer increases proportionally, and thermal-transfer inefficiencies become more significant. As a result, the additional energy input does not translate into a proportional increase in useful heating or cooling output. The dominant loss mechanisms are therefore the increased compressor work demand, associated electromechanical conversion losses, and reduced incremental heat-transfer benefit at elevated operating speeds. By the way, heat exchanger characteristics (e.g., fin density, surface area, refrigerant flow rate) influence the rpm–COP relationship. The nature of heat exchanger has a direct bearing on the extent to which any increase in compressor rpm is translated into productive thermal performance. The current system had a condenser that was an aluminum two-manifold single-pass heat exchanger, and the evaporator was set to a single-pass heat exchanger, with tubing run under the seat surfaces. These characteristics control the accessible heat transfer zone, refrigerant residence characteristics, as well as the thermal exchange between the refrigerant and the seat structure. A higher rpm can result in higher heat transfer during the initial rpm but beyond that point any contact area or geometrical constraints of an exchanger can cause diminished thermal utilization with the result that greater power input will result in a lower COP gain at high rpm.

### Data analysis and acquisition

Analyzed data from the car seat thermal management system were used to determine the primary components that contribute to the COP. Subsequently, predictive models were constructed using certain variables to forecast the COP. The experiments were carried out in a Renault Toros Station Wagon that was previously devoid of an HVAC system. The system was created by experimental modifications to the car components, including the addition of expanded evaporator coils under the surfaces of the driver seats. The frontal perspective of the system emphasizes crucial elements, including the electric motor, compressor, dryer, and condenser. On the other hand, the rear view displays the control panel, which includes a fan, inverter, electrical on–off indicators, ambient temperature thermometer, manometer for measuring compressor intake and outlet pressures, and a button for activating seat heating or cooling. The study gathered subjective feedback from a human participant and examined the relationship between temperature readings of seat surfaces and the thermal performance of the HVAC system, as documented in reference [21]. This study builds upon the authors’ prior research [20] by conducting comprehensive analysis using statistical and ML techniques, as well as developing prediction models. The variable that is reliant on other factors, known as the COP, is the focus of our models.

Throughout the testing process, data were gathered about the temperatures of the seat surface, the inlet and outlet temperatures of the HVAC equipment, and the pressures and speed at the inlet and outlet of the compressor. 23 thermocouples were used in total, with five thermocouples each for the seat back and cushion surfaces, and the remaining for the HVAC system units. The compressor mass flow rate and power intervals, along with the HVAC COP and fan power, are specified. The condenser and evaporator heat exchangers are described, and the vapor compression cycle commonly used in automotive applications [22] is mentioned. Mechanical and electrical conversion effects were incorporated into the energy analysis through the compressor power formulation. The electrical input to the motor was evaluated using the voltage, current, power factor, and motor efficiency, where the power factor was taken as 0.82 and the electric motor efficiency as 0.85. In addition, the HVAC system used DC fans with a nominal power of 20 W, and the compressor operated within a power range of 500–1000 W. Therefore, the reported energy assessment accounted for both compressor drive requirements and fan power consumption rather than relying only on ideal thermodynamic estimates. The assessment of uncertainty in sensor measurements is crucial for precise analysis. Uncertainties in experimental measurements are reported, ranging from 4% for compressor power to 1–1.5% for heat transfer calculations and 0.4% for COPs [20]. The augmented uncertainty in COP is attributed to systematic errors in sensor measurements.

### ANN model development

**Fig. 3:**
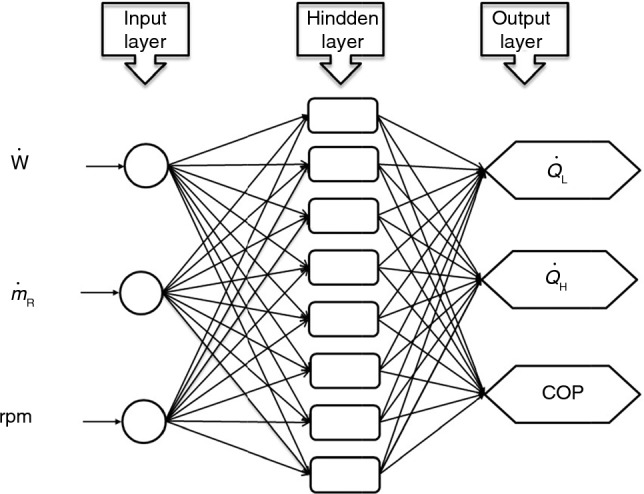
The configuration diagram of the developed MLP network model

An ANN model was developed to estimate the $\dot{Q}$_L_, $\dot{Q}$_H_, and COP values. The W, ${\dot{\mathrm{m}}}_{\mathrm{R}}$, and rpm values, namely work, mass flow, and revolution per minute, respectively, ​​were defined as input parameters in the input layer of the network model, which has a multi-layer perceptron (MLP) architecture, and the $\dot{Q}$_L_, $\dot{Q}$_H_, and COP values ​​were obtained in the output layer. The configuration diagram of the developed MLP network model is shown in Fig. 3. In determining the number of neurons in the hidden layer of MLP networks, the method applied in the literature was used.

An analysis was conducted on the performances of models with varying numbers of neurons in the hidden layer. Best performance was obtained with the model having 14 neurons in the hidden layer. The network model was trained with 24 data sets, 18 for training and 6 for testing. The MLP network model is trained using the training technique of Bayesian Regularization. The TanSig and Purelin transfer functions were used in the hidden and output layers of the model. The performance study of the constructed ANN model used typically employed performance parameters. The mathematical formulas for determining the MSE, *R*, and margin of deviation (MoD) as performance metrics are provided below [20]:

1 $${\mathrm{MSE}} = \frac{1}{N}\mathop \sum \limits_{\text i = 1}^{N} \left( {X_{{{\mathrm{targ}}\left( \text i \right)}} - X_{{{\mathrm{pred}}\left( \text i \right)}} } \right)^{2}$$

2 $$R = \sqrt {1 - \frac{{\mathop \sum \nolimits_{\text i = 1}^{N} \left( {X_{{{\mathrm{targ}}\left( \text i \right)}} - X_{{{\mathrm{pred}}\left( \text i \right)}} } \right)^{2} }}{{\mathop \sum \nolimits_{\text i = 1}^{N} \left( {X_{{{\mathrm{targ}}\left( \text i \right)}} - X_{{{\mathrm{av}}\left(\text i \right)}}} \right)^{2} }}}$$

3 $${\mathrm{MoD}} \left( \% \right) = \left[ {\frac{{X_{{{\mathrm{targ}}}} - X_{{{\mathrm{pred}}}} }}{{X_{{{\mathrm{targ}}}} }}} \right]x 100$$

While the developed ANN model demonstrates exceptional predictive fidelity within the experimental envelope, it is subject to the inherent limitations of data-driven architectures regarding extrapolation. Unlike mechanistic models based on first principles, ANNs function primarily as sophisticated interpolation engines that map nonlinear relationships within a bounded feature space. Consequently, predicting system performance such as COP or heat transfer rates, beyond the training boundaries of 500–1600 rpm or outside the investigated thermal loads may lead to increased uncertainty, as the model lacks the underlying physics to account for regime shifts or hardware limits not represented in the training data. For these reasons, the current model is optimized for the specific operational range of this modified HVAC architecture, and further data collection would be required to maintain generalizability in extreme or unprecedented operational conditions.

## Results and discussion

The thermal interaction between occupants and an automobile seat is essential for maintaining passenger comfort. A thorough experimental setup was established to investigate this, using heat exchanger coils from the HVAC system to control seat surface temperatures. The setup included a physical vehicle compartment with equipment and an observer for assessing passenger comfort. The experimental setup included a specially adapted automotive HVAC system for accurate data collection. Sensors were strategically placed around the configuration to measure key metrics such as airflow, seat surface temperatures and the overall performance of the HVAC system. The sensors continually gathered data, capturing the thermal behavior of the car interior under different operating conditions, thereby delivering valuable information to enhance passenger comfort. The collected data delivers an important information about the thermal behavior of the vehicle interior in different operating conditions, creating a basis for the evaluation of the system performance. The metrics obtained such as the airflow rates and the seat surface temperatures can be analyzed to establish correlations between the efficiency of the system and operational aspects such as the rotational speed (rpm) The following figures show in detail these connections. This gives a complete picture of how fluctuations in rpm. influence critical performance parameters such as the COP, power consumption and heat transfer properties. Such assessments are crucial for improving HVAC performance to achieve a compromise between passenger comfort and energy economy.

**Fig. 4:**
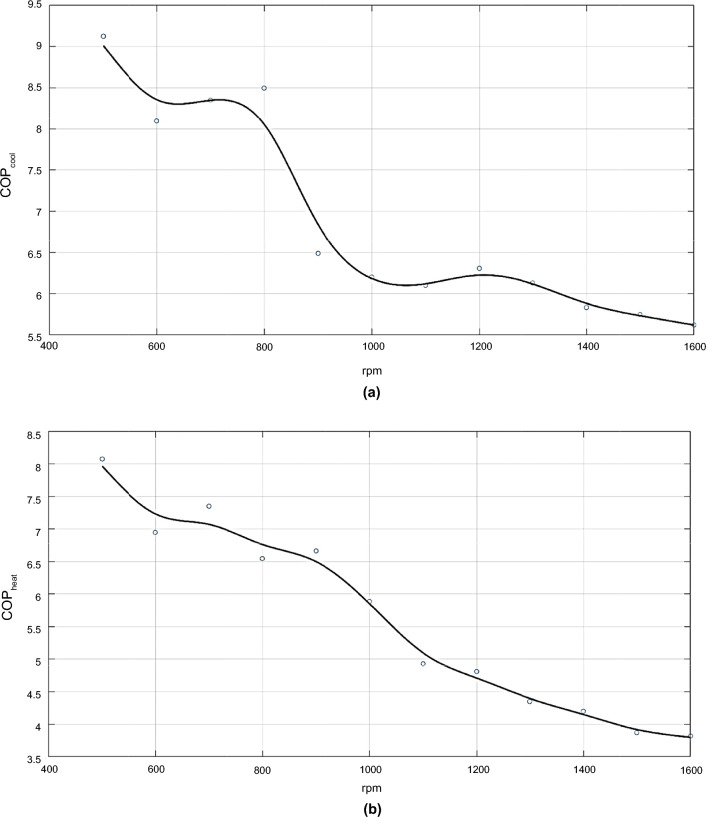
Alteration of COP with respect to rpm, **a** cooling and **b** heating

**Fig. 5:**
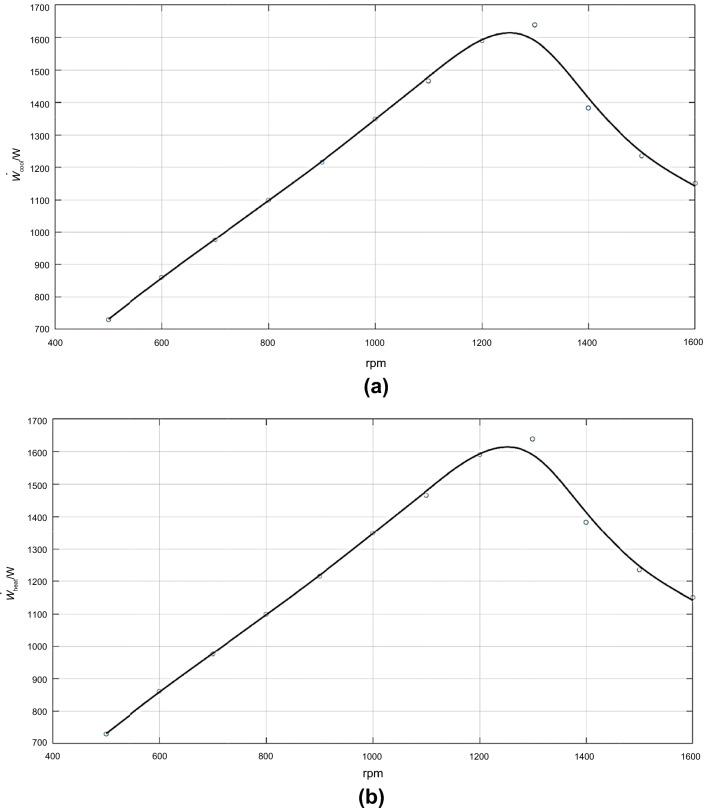
Alteration of W according to rpm, **a** cooling and **b** heating

**Fig. 6:**
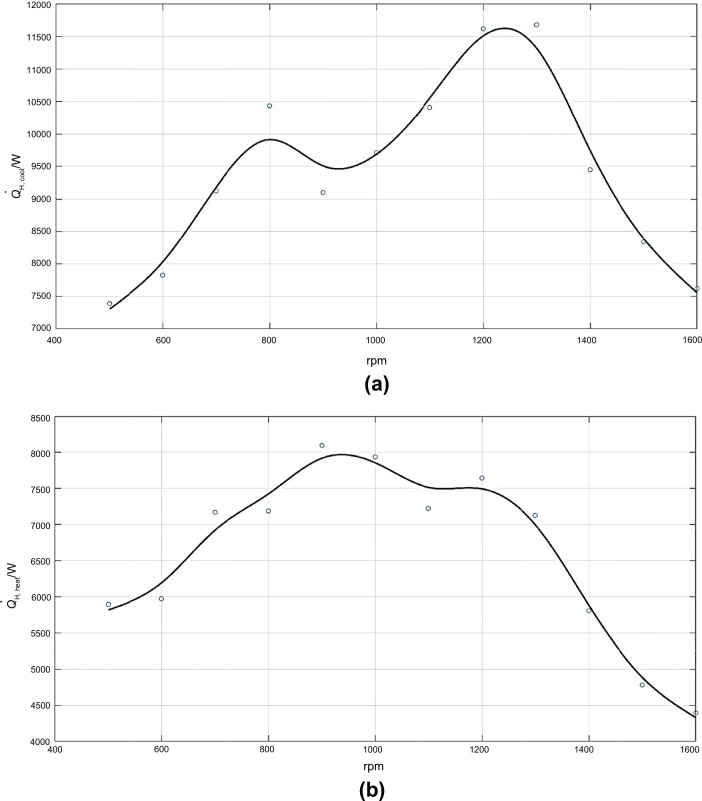
Alteration of *Q̇*_H_ with respect to rpm, **a** cooling and **b** heating

**Fig. 7:**
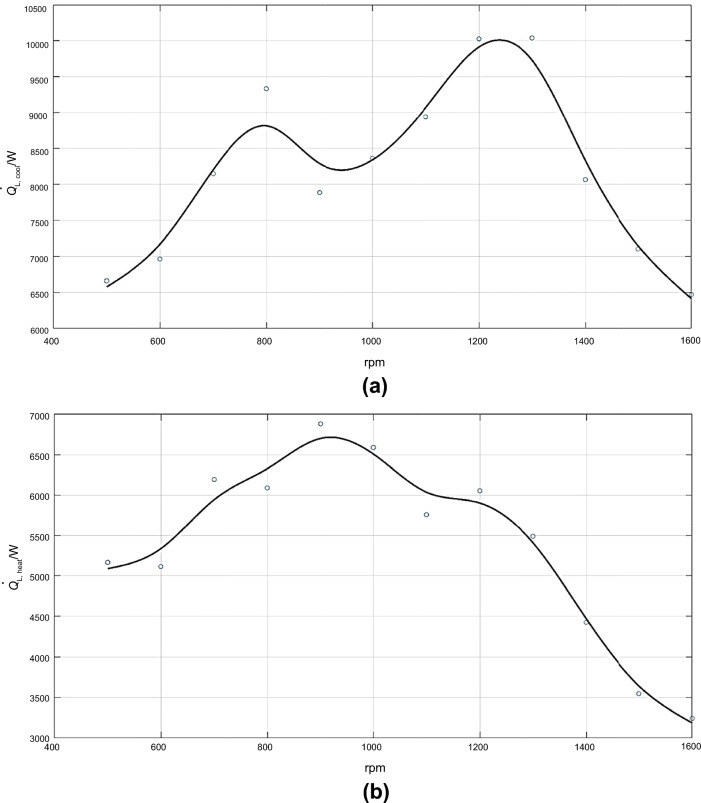
Alteration of *Q̇*_L_ with respect to rpm, **a** cooling and **b** heating

Figures 4-7 all indicate that there is a strong effect of compressor rotational speed on the thermodynamic behavior of the system during cooling and heating. The findings show that rpm and COP, power consumption, and heat transfer rates are highly nonlinear, showing that there is an optimal operating range, and no response with the increasing speed is continuously improving. Figure 4 shows the COP variations with rpm, which provides useful insights into the system efficiency under different operating situations. For example, in case of cooling (Fig. 4a), a higher rpm could have different effects on the COP for increasing it by improving the heat exchange rates and the distribution of the air flow or for decreasing it due to the increase in energy consumption and system inefficiencies. It is mainly based on the efficiency of the heat exchangers, thermal load in the vehicle cabin and the overall energy balance of the HVAC system.  Similarly, Fig. 4b studies heating operations where the effect of rpm changes on heating efficiency is considered. In heating mode, the increase of the rotating speed may improve the heat distribution and speed up the temperature distribution inside the cabin. Any such improvement has to be carefully balanced against the negative impacts, such as increased power consumption, increased mechanical wear and probable inefficiencies in thermal transfer systems. The figure illustrates the threshold beyond which further increments in rpm result in decreasing returns, indicating that greater energy input does not correspondingly improve heating efficiency. In summary, within the cooling mode, COP reaches its maximum value at low speed and decreases gradually with rise in rpm with slight recovery in the intermediate range. The same pattern is continued with heating mode with a significant decrease in COP between low and high speed. This decrease in COP at higher rates of rotational speed indicates that the increment of the compressor work is larger than the increment of the useful thermal effect. That is, even though increased speed improves the rate of circulation of a refrigerant, increased input power at high rpm decreases the energetic efficiency of the cycle. The findings hence show that low and moderate compressor speeds are more desirable to efficient operation of the system.

Figure 5 provides an analysis of power consumption in relation to the rpm. In cooling mode, as seen in Fig. 5a, a distinct relationship exists between rising rpm and a subsequent increase in power consumption. This trend shows an increased mechanical work required to sustain the higher cooling capacity and hence requires higher energy input. These results underscore the critical need for balancing operating velocity with energy efficiency in cooling systems. Figure 5b also investigates the relation between power consumption and rpm in heating mode. The data show a similar increasing trend for energy use as rpm increase. This insight underscores the need to enhance system performance to reduce excess energy use while ensuring adequate heating output. It can be concluded from the figures that during both cooling and heating cycles, the power consumption rises gradually with rpm to about 1250–1300 rpm after which a downward trend is recorded. The first rise is explained by high mechanical efforts to maintain high refrigerant mass flow speed and pressure rise across the compressor. The idea of the existence of a peak followed by a decrease with an increase in rpm would imply that the cycle is in such a state that further increase in speed does not proportionally raise the effectiveness of the compression. This could be related to the flow restrictions, rising irreversibilities or decreasing heat exchanger performance at high-speed operations. The fact that the trends of cooling and heating are nearly identical also shows that the speed of the compressor is a dominant influence that controls the input power irrespective of the operating mode.

Figure 6 examines the heat transfer characteristics of the system by evaluating fluctuations in *Q̇*_H_. In Fig. 6a, illustrating the cooling mode, *Q̇*_H_ denotes the heat discharged by the system into the environment. This metric is an important measure of a system's ability to dissipate excess thermal energy effectively, which is required for steady operation and to avoid overheating. The figure elucidates the effect of change in operating conditions on the cooling capacity of the system providing key information to improve the heat rejection. In contrast, Fig. 6b highlights the heating mode. Here, *Q̇*_H_ is the quantity of useful heat provided to the specified target. This shows the capability of the system to transfer energy efficiently for heating requirements. The patterns shown in Fig. 6b are analyzed to assess the system’s performance in meeting heating demands with a reduction in energy losses. The data provides a complete picture of the system’s thermal management, letting engineers optimize performance by balancing heat dissipation in cooling and heat application in heating. In heating mode, the useful heating output, *Q̇*_H_, shows a similar non-monotonic behavior with maximum rotational speed instead of the peak values at moderate speeds. This behavior indicates that the increase of the compressor speed is advantageous only until a certain limit, above which the thermal performance deteriorates. The decrease of *Q̇*_H_ at at high speed may be due to the insufficient residence time for heat transfer, the deterioration of the performance of the exchanger and the increase of thermodynamic losses in the cycle.

Figure 7 presents a complete insight on the variation of *Q̇*_L_, the heat extracted from a lower-temperature source, such as ambient air or an alternative medium, with respect to rpm. In cooling mode, as shown in Fig. 7a, *Q̇*_L_ is the system’s ability to extract heat from its surroundings. The ability of the cooling process to absorb heat is an important factor in determining overall efficiency. The variations  in *Q̇*_L_ with increasing rpm. show the extent of the system’s performance adjustment to different operating demands, revealing its efficiency and cooling capacity. In contrast, Fig. 7b, however, shows the heating mode. The variations in *Q̇*_L_ as a function of rpm, show the balance of energy absorbed and released by the system to keep the heating efficiency. In sum, in cooling mode, the cooling-side heat absorption increases with rpm up to an optimum region and then declines sharply as the speed is further increased. In heating mode, *Q̇*_L_ also rises to a maximum at moderate speed before falling at high rpm. These findings confirm that both the evaporator-side and condenser-side thermal interactions are strongest within an intermediate speed range. At very high rpm, the cycle appears unable to maintain effective heat absorption and rejection simultaneously, leading to lower thermal capacity and poorer overall performance. This observation is fully consistent with the decline in COP reported in Fig. 4.

Combining the findings, the system cannot be run at full compressor speed all the time provided the aim is to get the maximum efficiency. The rpm is initially beneficial in augmenting thermal capacity, but only over a certain operating range. Outside this range, an increase in compressor work and the decline in the effectiveness of heat transfer result in a decline in COP and thermal performance. Based on this, the intermediate speed range seems to offer the most appropriate tradeoff between nominal capacity in cooling and heating operations as well as energy efficiency. The significance of these results is in the control of the functioning of systems and optimization of their operation since it indicates that it is necessary to choose the compressor speed not only with the current load requirement but also with the thermodynamic penalty in the case of high rotational speed.

By evaluating data identifying complex patterns and creating accurate prediction models by ML, a branch of AI offers a fresh approach to investigating these effects. For complex systems like HVAC and vehicle thermal comfort where the traditional method of physics-based calibration takes a long time, ML is particularly effective. To find links between inputs and outputs They are especially employed when the basic mechanisms of system parameters are poorly understood. ML techniques are regression optimization and neural networks. The ANN model provides a better understanding of the efficiency and operation of the vehicles thermal management system. This allows engineers to improve design, improve passenger comfort and to simulate different operating scenarios. An ANN model is developed in this work to predict the important  parameters like the *Q̇*_L_, *Q̇*_H_, and COP. The model was trained with data on temperature variations system responses to different conditions and thermal distribution over the car seat. As a result, the model was able to learn complex patterns and generate accurate predictions regarding heat transfer in both heating and cooling modes. Additionally, the model helps to balance comfort and energy use by projecting the COP which helps to assess the system’s energy efficiency. This tool makes it easier to create advanced energy-efficient car heating and cooling systems.

**Fig. 8:**
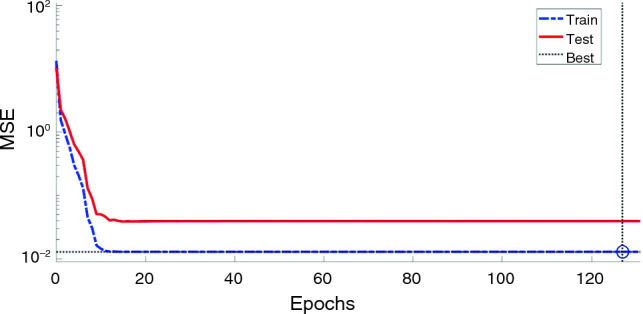
The training performance graph for the MLP model

As seen in Fig. 8, a training performance graph was created using training process data to assess the neural network models training phase. The model’s training progress is shown in this graph with special attention paid to the MSE values. The high MSE values at the beginning of the training phase indicate a bigger difference between the expected and the actual outputs. The MSE values, however, decrease gradually with training and the internal parameters of the model are improved. The gradual decrease suggests that the model is getting better in accuracy as it learns from the data. The training phase ends when the MSE values of the test and training data sets converge to an optimal level, which means that the model has performed at its best. This confirms that the network has learned the patterns in the data successfully, neither over- nor underfitting. The results show how robust the model is to handle complex relationships and how ready to generate accurate predictions on new data. After the training phase verification, a detailed analysis of the developed ANN model prediction performance was carried out. The ANN models predicted values were compared to the actual target values to assess the model’s accuracy in predicting three important parameters: *Q̇*_L_, *Q̇*_H_, and COP.

**Fig. 9:**
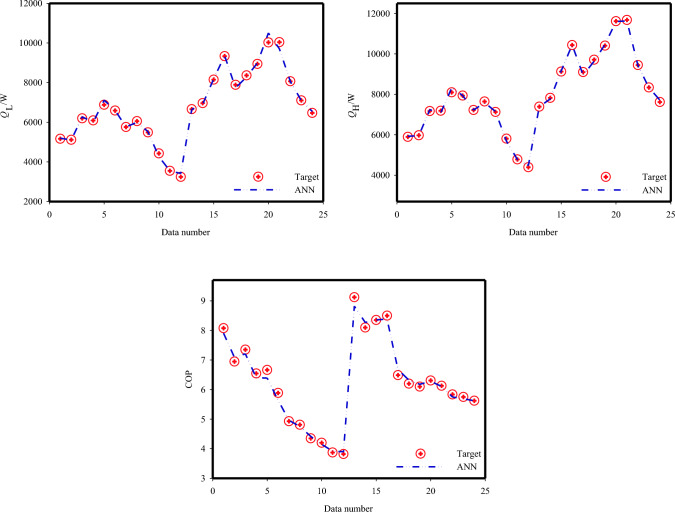
Comparisons of the predicted and the target values

Figure 9 shows a graphic representation of these comparisons. The ANN models predictions closely match the target values indicating high predictive accuracy according to the graphs for each parameter. The model has been able to reflect the underlying relationships in the data with very small deviations which can be used for accurate allowing for accurate *Q̇*_L_, *Q̇*_H_, and COP forecasts. Such high accuracy guarantees the dependability of the ANN models for real world applications particularly for predicting thermal behavior under different conditions. Its ability to repeat real-world results consistently demonstrates how effective it is at optimizing vehicle thermal management while improving  occupant comfort and energy efficiency. Overall, the findings support the model’s strong prediction abilities which qualify it for sophisticated thermal management analysis.

**Fig. 10:**
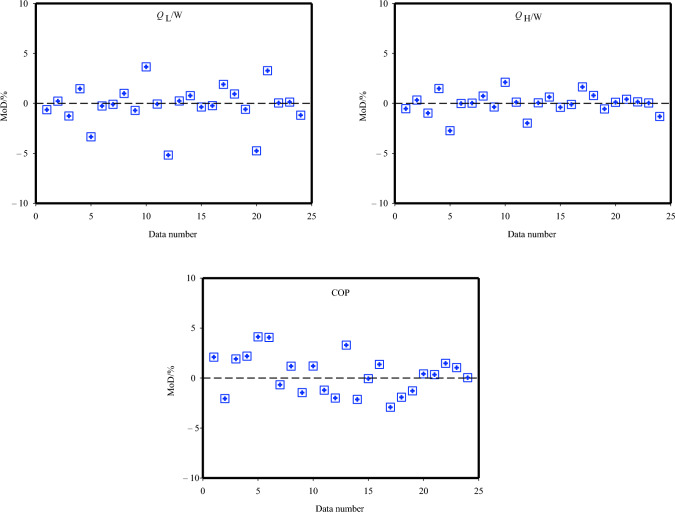
The deviations between the *Q*_L_, Q_*H*_, and COP values ​​obtained from the ANN models and the target values

Figure 10 shows the calculated differences between the actual target values and the ANN models predicted values for *Q̇*_L_, *Q̇*_H_, and COP. The deviation values for every data point are shown in these graphs indicating how near the zero-deviation line the data points are. This nearness suggests that the model’s prediction errors are small. A small percentage of data points with notable deviations are visible in the graphs demonstrating the model’s high degree of prediction accuracy. For *Q̇*_L_, *Q̇*_H_, and COP the corresponding average deviation values were determined to be − 0.21, − 0.02, and 0.38%. The results show the excellent ability of the model to predict these vital parameters with very small errors. This small deviation is required to validate that the ANN model can correctly  and consistently predict *Q̇*_L_, *Q̇*_H_, and COP values, and thus, it confirms the efficacy of the ANN model for the prediction of the thermal behavior. The low error rates also indicate that the ANN model is a reliable tool for real-world vehicle thermal management applications as it has successfully captured the intricate relationships between the input variables. These results highlight how well the model predicts system performance in a consistent and accurate manner.

**Fig. 11:**
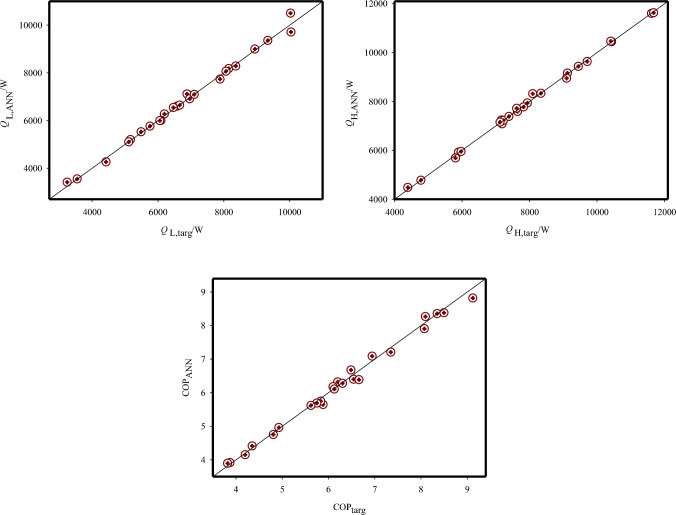
The target and the predicted values

The ANN models predicted values are displayed on the y-axis in Fig. 11 while the target values for *Q̇*_L_, *Q̇*_H_, and COP are plotted on the x-axis. It is evident from looking at the data points that most of them fall near the zero-error line suggesting that the model’s predictions are highly accurate. An extremely low average error between the predicted and actual values is shown by the ANN models computed MSE of 1. 29E-02. The model’s predictions and the desired values also showed an almost perfect correlation as indicated by the *R* which was determined to be 0.99659. The ANN model with a high R value has successfully captured the underlying relationships between the input and output variables, ensuring that its predictions are close to the real outcomes. The results show that the developed ANN model can predict *Q̇*_L_, *Q̇*_H_, and COP values with a low degree of error. The model is an efficient tool for forecasting and evaluating the thermal performance of vehicle systems because of its low MSE and high *R* values which attest to its high reliability. This degree of accuracy is necessary for system design optimization and for enhancing occupant comfort and energy efficiency in real-world automotive applications. All things considered the findings offer compelling proof that the ANN model predicts the important thermal parameters with optimal accuracy.

## Conclusion

Thermal interaction between passengers and car seats is vital for comfort. An experiment utilizing HVAC heat exchanger coils was designed to adjust seat surface temperatures, showcasing a novel methodology. The setup involved a vehicle compartment prepared for passenger comfort observation, sustained by a precisely configured automotive HVAC system. The study shows that ML is well-suited to complex systems, such as HVAC and vehicle thermal comfort, especially when physics-based calibration is time-consuming. The MLP network model is used to connect inputs and outputs, which is particularly useful when the system parameter processes are not clear, with the Bayesian Regularization training technique. The ANN model gives an insight into the thermal management system of vehicle efficiency and operation. The developed ANN model showed a high accuracy in the prediction of key parameters like *Q̇*_L_, *Q̇*_H_, and COP, confirming its potentiality as a reliable tool for optimizing automotive HVAC. This model utilizes thermal data and predicts the performance of the system to facilitate the design of energy-efficient climate control systems for better passenger comfort. Future research could incorporate complex ML models and real-time data integration. Generalizability of the model to other vehicle types and HVAC configurations can be improved by expanding experimental setups with larger datasets. Below are the key findings of the analysis.

- Rpm increases heat exchange rates and airflow dispersion, but it increases energy consumption and system inefficiencies, which reduces COP. In heat mode, increasing the rpm will improve the dispersal of heat and temperature but will also increase energy consumption, mechanical wear and thermal inefficiencies. After a certain point, increases in rpm have diminishing returns, meaning the input of energy no longer increases efficiency.
- Power consumption increases with RPM in cooling and heating modes due to more mechanical work. The heat transfer capacities of the system are indicative of its ability to dump excess heat during cooling and to deliver useful heat during heating. Good heat dissipation can keep the device working steadily and prevent it from overheating, and good energy transfer can meet the heating demand and reduce loss.
- The variation in *Q̇*_L_, during the cooling mode, affects the system’s efficiency and cooling capacity due to increased rpm, but in heating mode, variations in *Q̇*_L_ indicate the balance between energy absorption and output required to maintain heating efficiency.
- The MSE was high in the beginning during the training phase, but it constantly decreased and reached the optimal levels for both training and test datasets in the end. The ANN model yielded an MSE of 1.29E-02, indicating low prediction error.
- The predicted values for *Q̇*_L_, *Q̇*_H_, and COP were very close to the target values, indicating high prediction accuracy. The correlation between the predicted and actual values indicated that the ANN model can successfully predict the values of *Q̇*_L_, *Q̇*_H_, and COP.
- The differences between predicted and actual values were small, with average differences of − 0.21% for *Q̇*_L_, − 0.02% for *Q̇*_H_, and 0.38% for COP, which showed low errors..
- The *R* was 0.99659, indicating that the predicted and actual values fit almost perfectly.
- These results validate the ANN model for the prediction of *Q̇*_L_, *Q̇*_H_, and COP with high accuracy, low error, and high correlation and can be used as a robust tool for the prediction of thermal performance prediction.
- Thermal management at seat level by the HVAC system in the automotive sector can improve passenger comfort and energy savings. Better cooling, heating, and COP prediction under diverse operating conditions can be achieved with the ANN framework in future vehicle climate-control systems.

## Data Availability

Data will be made available on request.

## List of symbols

AI: Artificial intelligence
ANN: Artificial neural network
COP: Coefficient of performance
HVAC: Heating, ventilation, and air-conditioning
$\dot{m}$: Mass flow rate, kg s^−^^1^
ML: Machine learning
MLP: Multi-layer perceptron
MoD: Margin of deviation
MSE: Mean squared error
*R*: Correlation coefficient
*Q̇*_H_: Heat rejected from the system, W
*Q̇*_L_: Heat extracted from the system, W
rpm: Revolutions per minute
*W*: Mechanical work of compressor, W
